# Identification of short-form RON as a novel intrinsic resistance mechanism for anti-MET therapy in MET-positive gastric cancer

**DOI:** 10.18632/oncotarget.5816

**Published:** 2015-10-26

**Authors:** Zheng Wu, Zhe Zhang, Xiaoxiao Ge, Ying Lin, Congqi Dai, Jinjia Chang, Xinyang Liu, Ruixuan Geng, Chenchen Wang, Huan Chen, Menghong Sun, Weijian Guo, Jin Li

**Affiliations:** ^1^ Department of Medical Oncology, Fudan University Shanghai Cancer Center, Shanghai 200032, China; ^2^ Department of Oncology, Shanghai Medical College, Fudan University, Shanghai 200032, China; ^3^ Department of Pathology, Fudan University Shanghai Cancer Center, Shanghai 200032, China

**Keywords:** drug resistance, RON, MET, gastric cancer, targeted therapy

## Abstract

Despite the promising results from initial studies, there are significant limitations in the application of MET-targeted therapy in gastric cancer. Intrinsic resistance is one of the major obstacles. The aim of this study is to identify the responsible receptor tyrosine kinases (RTKs) that determine the unresponsiveness of MET inhibitor in MET-positive gastric cancer. through an RNA-interference-based functional screen targeting most human RTKs, we identified that activation of the fibroblast growth factor receptor 2 (FGFR2) and recepteur d'origine nantais (RON) pathways attenuated MET inhibitor-induced suppression of cell proliferation and migration. Notably, in the two forms of RON pathway activation, only upregulation of short-form RON (sf-RON), but not stimulation of full length RON with macrophage stimulating protein, conferred MET inhibitor resistance *in vitro* and *in vivo*. Furthermore, the profile of the gastric cancer samples observed that sf-RON was frequently upregulated in MET-positive gastric cancer. Our findings indicate that activation of the sf-RON signaling pathway represents a novel mechanism underlying MET inhibitor unresponsiveness. A combination strategy with drugs targeting both RON and MET pathways is believed to improve the efficacy of MET-targeted therapy.

## INTRODUCTION

MET, as a member of receptor tyrosine kinase (RTK) family, plays a causal role in tumor cell survival, growth, angiogenesis and metastasis [1]. *MET* gene amplification has been reported in 1.5 to 10.2% of gastric cancers (GCs), and overexpression of MET can be detected in more than 20% of GC samples [2–6]. Excessive activation of the hepatocyte growth factor/scatter factor (HGF/SF)-MET axis is considered to correlate with poor prognosis and drug resistance in various human cancers, including GC [1, 2, 7, 8]. Thus, anti-MET has emerged as an attractive strategy to treat patients with GC that harbors dysregulated HGF/SF-MET signaling. During the past decade, monoclonal antibodies and small-molecule tyrosine kinase inhibitors targeting the HGF/SF-MET pathway have been evaluated in preclinical experiments and clinical trials [9].

Initial results from most studies suggest that anti-MET therapy is able to improve overall survival (OS) and progression-free survival (PFS) in several cancer types [9]. Recently, a double-blind and randomized phase 2 clinical trial showed that the addition of rilotumumab, a fully human monoclonal antibody of HGF, to chemotherapy improved the prognosis in patients with GC or esophagogastric junction cancer, especially in MET-positive (MET+) subgroup. However, in MET+ subgroup, the objective response rate to the treatment of rilotumumab plus chemotherapy was only 50% [10]. Moreover, clinical trials studying the efficacy of MET-targeted tyrosine kinase inhibitors (TKIs) as monotherapy in patients with metastatic gastric adenocarcinoma only showed minimal efficacy, even in MET-amplified subgroup [11, 12]. Limited response rate and minimal efficacy suggested that a proportion of patients with MET+ stomach tumors may be insensitive to MET targeted therapy. Intrinsic resistance to MET inhibitors emerges as a major limitation in the application of MET-targeted therapy in gastric cancer.

In order to improve the efficacy of MET-targeted therapy, understanding the potential molecular mechanisms of intrinsic resistance to MET inhibitors is imperative. Activation of compensatory pathways is recognized as an important sign of resistance to targeted therapies [13, 14]. Despite inhibition of the drug targets, the presence of bypass RTKs that maintain the activation of downstream signaling pathways results in failure of targeted therapies [15]. In this study, we conducted a functional screening with a small interfering RNA (siRNA) library targeting most human RTKs, to identify bypass tracks that affect the responsiveness of MET+ GC to anti-MET agents. The results of screening and subsequent validation revealed that activation of fibroblast growth factor receptor 2 (FGFR2) and recepteur d'origine nantais (RON) pathways attenuated MET inhibitor-induced suppression of cell proliferation and migration. RON pathway was identified to promote resistance to anti-MET agents for the first time. As another member of the MET proto-oncogene family [16], RON has been found to be activated aberrantly in various malignances, including GC [17], and is closely related to MET [1, 18]. Two transcripts of this gene coding a full-length RON (fl-RON) and a short-form RON (SF-RON) have been detected in GC tissues [19]. Here, we found that in the two forms of RON pathway activation, upregulation of sf-RON, but not stimulation of f-RON with macrophage stimulating protein (MSP), conferred MET inhibitor resistance. We also found that sf-RON was up-regulated in MET+ GC. On the basis of these findings, MET/RON dual inhibition might be necessary for treating MET+ GC patients with RON pathway activation.

## RESULTS

### siRNA screening identifies RTKs influencing the sensitivity of GC cells to PF

**Figure 1:**
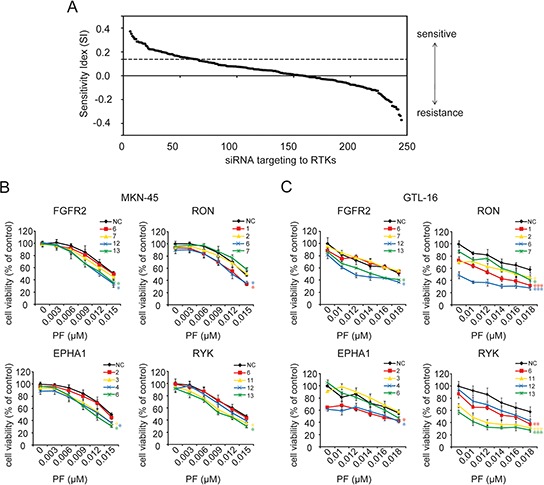
siRNA library screening identified gene candidates that could be targeted to sensitize GC cells to PF **A.** The combined effect of siRNA and PF was assessed using the SI. Positive SI scores represented the sensitizing effect, and negative SI scores indicated antagonism to the treatment. In this study, SI scores in the upper quartile (above dashed line) were considered as conferring a strong drug-sensitizing phenotype. **B.** and **C.** Silencing of EPHA1, RYK, RON, or FGFR2 showed significant enhancement of PF effectiveness. MKN-45 and GTL-16 cells were transfected with non-targeting control (NC) siRNA and 4 siRNA species targeting each gene. Cell viability was measured using the CCK-8 cell-proliferation assay after 4 days of drug exposure. The percentage of viable cells is shown relative to untreated non-targeting controls. Values are the mean ± SD of 3 replicates; *, *P* < 0.05, **, *P* < 0.01, ***, *P* < 0.001, compared with the PF-treated NC group.

Firstly, to identify RTKs whose knockdown selectively sensitizes GC cells to MET inhibitor, a highly selective MET inhibitor PF and a MET-addicted GC cell line MKN-45 were chosen. This cell line is deemed to harbor MET gene amplification and stably over express MET [20]. Our results indicated that MET+ MKN-45 cells were much more responsive to PF than the other MET- GC cell lines, with an IC50 of 0.018 μM (Supplementary Figure S1A and S1B). Then, MKN-45 cells were transfected with a siRNA library targeting 60 human RTK genes, with each gene targeted by 4 individual siRNAs. The combined effect of siRNA and PF was assessed using the SI. As mentioned previously [21], positive SI scores represented the sensitizing effect. All SI scores of 240 siRNA hits were ranked (Figure 1A), and RTKs with scores in the upper quartile were extracted for the subsequent analysis.

To avoid off-target effects, those genes with only one SI score in the first quarter were rejected. The remaining 16 candidates were re-assessed through cell proliferation inhibition assay. As a result, only knockdown of FGFR2, RON, Eph receptors A1 (EPHA1), and receptor-like tyrosine kinase (RYK), but not the other 12 RTKs significantly enhanced the effectiveness of PF in MKN-45 cells (Figure 1B and Supplementary Figure S2). The synergistic effect of silencing these 4 RTKs with PF was further confirmed in another MET-addicted GC cell line GTL-16 (Figure 1C). Together, these results suggested that multiple RTK pathways tend to influence the responsiveness of GC cells to MET inhibiton.

### B-FGF induced FGFR2 activation attenuates PF-induced growth inhibition and motility suppression

**Figure 2:**
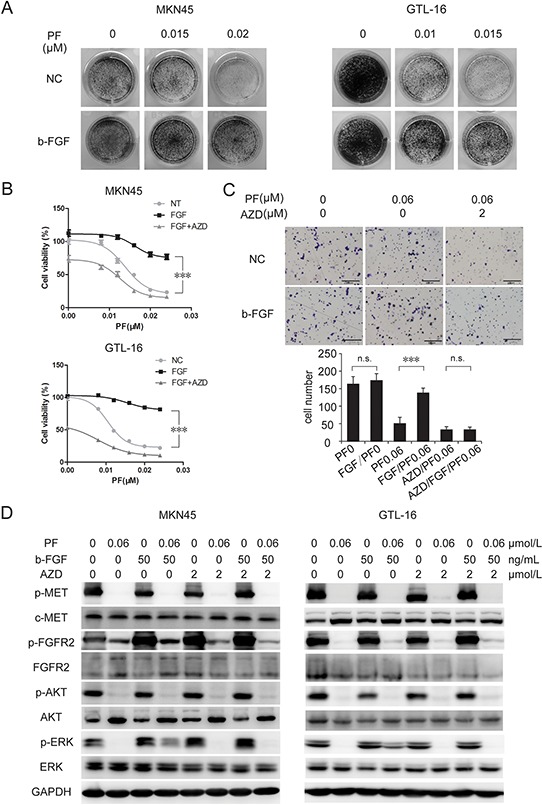
B-FGF-induced FGFR2 activation promoted PF unresponsiveness in MET-addicted GC cell lines **A.** b-FGF induced PF resistance in MET-amplified cancer cell lines. Colony formation assays showed that stimulation of b-FGF resulted in obvious increase in PF-resistant cell clones. **B.** Combination of AZD4547 and PF significantly increased growth inhibition of MET-amplified cancer cells. MKN-45 and GTL-16 cells were exposed to PF (0–0.024 μM) or a combination of PF and b-FGF (50 ng/mL) or a combination of PF/b-FGF and AZD4547 (5 μM). Cell viability was measured using the CCK-8 cell-proliferation assay after 3 days of drug exposure. Values are the mean ± SD of 3 replicates. **C.** Activation of FGFR2 signaling pathway attenuated PF-induced suppression of cell motility. Transwell migration assays of GTL-16 cells were performed with the treatment of PF (0.01 μM), b-FGF 50 (ng/ml) and AZD4547 (2 μM) for 48 hours. The results are representative images of migration assays, Bars, 50 μm (top), and quantification of cells (bottom). Values are the mean ± SD of 3 assays; ***, *P* < 0.001, n.s., no statistical significance. **D.** b-FGF conferred PF resistance by restoring ERK activation. Cells were pretreated with TKIs for 6 hours, and then stimulated with b-FGF for 30 minutes before whole cell lysates were collected. Western blot showed that ERK, but not AKT, reactivated after adding b-FGF to the treatment of PF.

**Figure 3:**
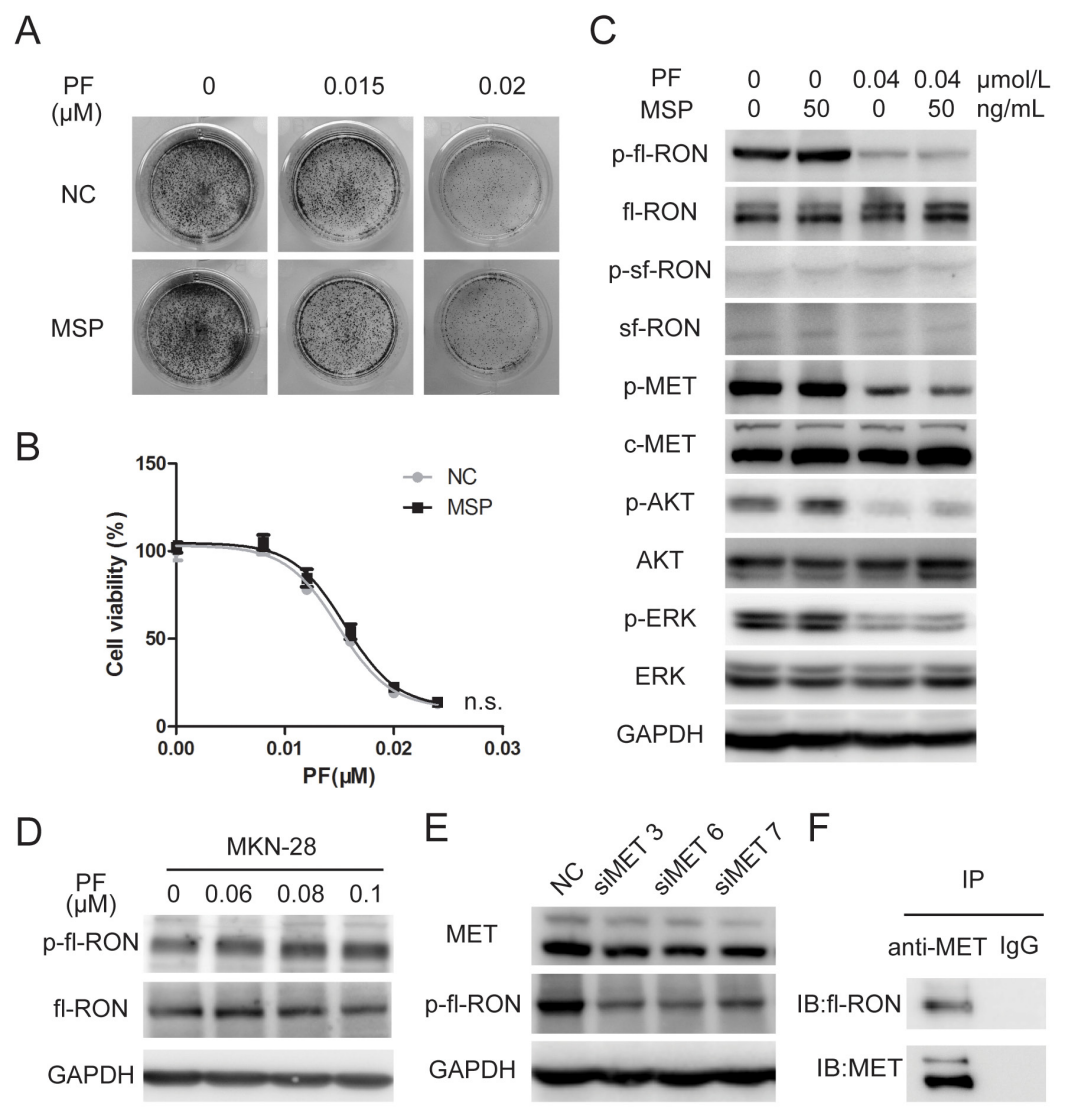
MSP did not contribute to PF unresponsiveness in MET-addicted GC cell lines **A.** Colony formation assays showed that stimulation of b-FGF resulted in no increase in PF-resistant MKN-45cell clones. **B.** MSP hardly influence growth inhibition of MET-amplified cancer cells by PF. GTL-16 cells were exposed to PF (0–0.024 μM) or a combination of PF and MSP (50 ng/mL). Cell viability was measured using the CCK-8 cell-proliferation assay after 3 days of drug exposure. Values are the mean ± SD of 3 replicates. **C.** MSP failed to rescue *p*-fl-RON from PF exposure. MKN-45 cells were pretreated with PF (0.04 μM) and MSP (50 ng/mL) before whole cell lysates were collected. Western blot analysis was conducted to examine the status of *p*-RON, *p*-MET, *p*-AKT, and *p*-ERK. **D.** PF did not inhibit phosphorylation of fl-RON in cell line with low expression of MET. MKN-28 cells were pretreated with PF (0.06–0.1 μM), then Western blot analysis was conducted to examine the status of RON and *p*-RON. **E.** Knockdown of MET weaken phosphorylated fl-RON. MKN-45 cells were transfected with 20 nM 3 distinct siRNA species targeting MET or NC siRNA. MET and phosphorylated fl-RON was measured by Western blot 48 hours later. **F.** Co-immunoprecipitation indicated that MET and fl-RON formed heterodimers in MKN-45 cells. Irrelevant IgG serves as a negative control.

In order to verify whether activation of EPHA1, RON, RYK, or FGFR2 pathways would contribute to PF resistance, we treated MKN-45 cells with PF (0–0.02 uM) and corresponding ligands of each receptor for 10 days. The colony formation assay revealed that b-FGF-induced activation of FGFR2 resulted in an obvious increase in PF-resistant cell clones (Figure 2A). Conversely, no detectable disparity was observed between the control group and the other 3 ligand-treated groups (Figure 3A and Supplementary Figure S3A). We next confirmed the contribution of FGFR2 to PF resistance in GTL-16 cells. And the addition of AZD4547, an FGFR inhibitor [22], to PF/b-FGF treatment restored PF-mediated proliferation inhibition (Figure 2B).

The activated MET signaling pathway not only promotes tumor cells proliferation, but also facilitates cell migration and invasion, which contributes to tumor metastasis [23–25]. Inhibition of MET through TKI or RNA interference was able to reduce lymph nodes or distant metastasis [26, 27]. By using transwell migration assay, we confirmed that PF markedly suppressed GTL-16 cell motility. Although treatment of b-FGF (50 ng/mL) alone could not enhance cell migration, b-FGF was able to nullify the motility suppression induced by PF. AZD4547 could neutralize the resistance to PF induced by b-FGF (Figure 2C). Western blotting showed that addition of b-FGF to PF treatment partially restored the *p*-ERK level, but not the *p*-AKT level, And adding AZD4547 to PF/b-FGF treatment suppressed p-ERK again (Figure 2D). These findings indicated that activation of FGFR2 signaling pathway rescued MET+ GC cells from PF sensitivity, significantly.

### MSP fails to reactivate fl-RON in the presence of PF

RON and MET belong to the same subfamily. It is clear that RON actively crosstalks with MET, and takes part in the regulation of tumorigenic activity [18]. Our previous data showed that knockdown of RON sensitized GC cells to PF. Unexpectedly, treating with MSP, the ligand of fl-RON, resulted in no increase of PF-resistant MKN-45 cell clones (Figure 3A). Cell viability assay in GTL-16 cells showed that MSP did not affect PF-induced inhibition of cell growth, neither (Figure 3B). Then we detected the phosphorylation status of RON and downstream signaling molecules with the treatment of PF and MSP. The western blot assay revealed that PF at 0.04 μM not merely blocked activation of MET, but also resulted in decreased fl-RON phosphorylation (Figure 3C). It is intriguing that PF, regarded as a highly selective inhibitor to MET, could cause decreased fl-RON phosphorylation. We performed a series of experiment to investigate that whether PF targets fl-RON directly or PF cause decreased fl-RON phosphorylation indirectly through other mechanisms. As shown in Figure 3, PF (0–0.1 μM) treatment in MKN-28 cells, a cell line with weak MET expression (Supplementary Figure S1), showed no inhibition to phosphorylation of fl-RON (Figure 3D). However, silencing MET via siRNA resulted in decreased fl-RON phosphorylation in MKN-45 cells (Figure 3E). This suggested the inhibition of PF to fl-RON may be depending on the status of MET. Co-immunoprecipitation analysis indicated that RON and MET could form heterodimers in GC cells, suggesting a transactivation of RON by MET (Figure 3F). MSP was unable to reactivate full-length RON and its downstream signaling when they were suppressed by PF (Figure 3C). Taken these data together, we concluded that in MET-addicted GC cells, fl-RON phosphorylation was inhibited by PF indirectly, probably through the inhibition of transactivation of RON and MET. And stimulation with MSP could not rescue the inhibition cause by PF.

### Upregulation of sf-RON impairs PF-induced inhibition of growth and migration in MET-addicted tumor cells

**Figure 4:**
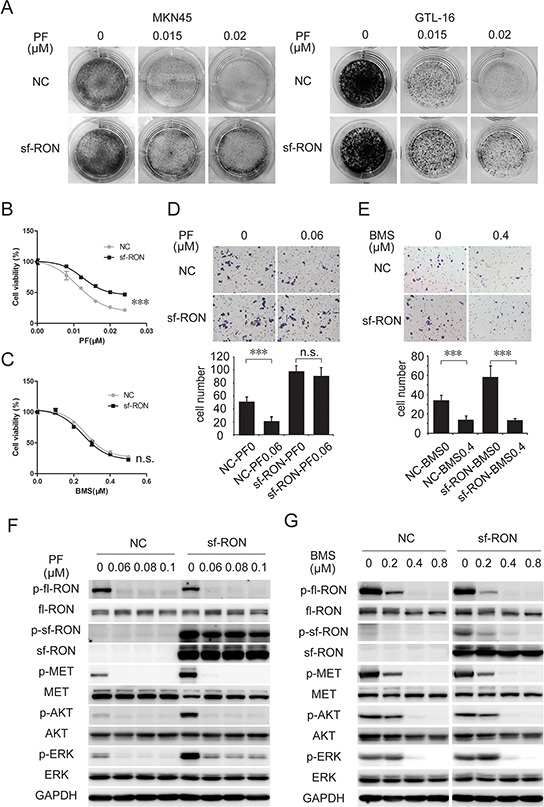
Upregulation of sf-RON attenuated PF-induced inhibition of cell proliferation and motility **A.** and **B.** sf-RON induced PF resistance in MET-amplified GC cell lines. A, Colony formation assays showed that PF-resistant cell clones in the sf-RON group were greater than those in the NC group. B, cells were treated with increasing doses of PF (0–0.024 μM), followed by CCK-8 cell-proliferation assay at 72 hours. **C.** MET/RON dual inhibition suppressed viability of cells with sf-RON overexpression effectively. Cells were treated with BMS (0–0.5 μM), followed by CCK-8 cell-proliferation assay at 72 hours. **D.** and **E.** BMS, but not PF, abrogates sf-RON-induced extra cell motility. Transwell migration assays of GTL-16 cells were performed with the treatment of 0.06 μM PF (D) or 0.4 μM BMS (E) for 48 hours. Representative images of migration assays and statistics in bar graphs as indicated. Bars, 50 μm. Values are the mean ± SD of 3 assays; ***, *P* < 0.001, n.s., no statistical significance. **F.** sf-RON confers PF resistance by restoring ERK activation. **G.** BMS total blocked phosphorylation of MET/RON and downstream signaling. Cells in the NC group and sf-RON group were pretreated with PF (F) or BMS (G) for 6 hours before whole cell lysates were collected. Western blot analysis was conducted to examine the status of *p*-RON, *p*-MET, *p*-AKT, and *p*-ERK.

MSP stimulation is not the only cause of RON pathway activation. Compared with fl-RON, sf-RON lacks most of the extracellular part. Thus, sf-RON proteins form constitutively active dimmers without the requirement for MSP [16]. Through Western blot assay, we noticed that phosphorylation of sf-RON was not influenced by PF (Figure 3C). To prove our conjecture that sf-RON contributes to PF resistance, the growth inhibitory effects of PF on sf-RON overexpressed cells and control cells were assessed through colony formation assay and cell proliferation inhibition assay. As shown in Figure 4A and Supplementary Figure S4A, in a panel of previously defined MET amplified cancer cell lines MKN-45, GTL-16 and EBC-1 [20, 28, 29], upregulation of sf-RON resulted in increased numbers of PF-resistant colonies. The inhibition rate was reduced significantly in sf-RON overexpressed groups compared with control groups (*P* < 0.001) (Figure 4B and Supplementary Figures S4B). Western blot analysis demonstrated that, when exposed to PF, *p*-AKT and *p*-ERK were blocked completely in control group cells, while *p*-ERK was sustained at a certain level in sf-RON overexpressed GTL-16 cells, and both *p*-AKT and *p*-ERK was sustained at a certain level in sf-RON overexpressed EBC-1 cells (Figure 4F and Supplementary Figure S4F).

Similar to MET, RON is also implicated in tumor invasion and metastasis [30, 31]. Sf-RON expression increased motility of tumor cells [19]. Therefore, we assessed the potential effect of sf-RON on PF-induced motility suppression. *In vitro* transwell assay demonstrated that sf-RON overexpression remarkably enhanced the ability of GTL-16 cells to move through filter micropores. As expected, motility of these sf-RON overexpressed cells was not affected by PF at 0.06 μM, while migration of cells in the control group was significantly suppressed (Figure 4D). Similar to GTL-16 cells, sf-RON expressing in EBC-1 weakened PF induced suppression of cell motility (Supplementary Figure S4D).

To overcome PF resistance induced by sf-RON, we tested the efficacy of BMS777607, a small molecular inhibitor targets both RON and MET [32], on sf-RON-overexpressed GTL-16 and EBC-1 cells. Through proliferation inhibition assay, we found that the sensitivity of sf-RON cells to BMS777607 was consistent with that of NC cells (Figure 4C and Supplementary Figure S4C). Transwell assay revealed that the excessive motility triggered by sf-RON was significantly abrogated by BMS777607 (Figure 4E and Supplementary Figure S4E). Indeed, inhibiting sf-RON activation by BMS777607 significantly restored PF effectiveness on proliferation and migration in MET+ cells, which were associated with their further blocking the downstream AKT and ERK signaling (Figure 4G and Supplementary Figure S4G). Taken together, these results implied that sf-RON pathway serves as a signaling compensatory mechanism, conferred resistance to MET inhibition.

### Upregulation of sf-RON contributes to PF resistance *in vivo*

**Figure 5:**
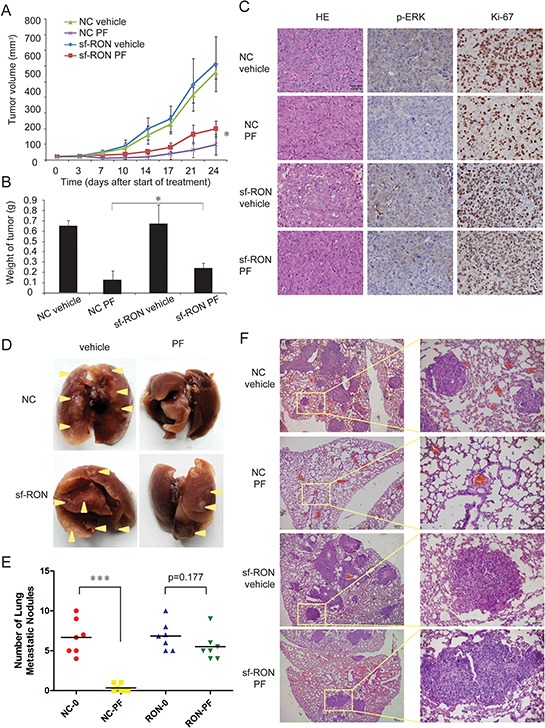
Upregulation of sf-RON contributed to PF resistance *in vivo* **A.** Xenograft tumors on male nude mice were established with sf-RON-overexpressed GTL-16 cells and NC GTL-16 cells. Mice were treated for 7 days/week with either vehicle control (normal saline) or 4 mg/kg PF by oral gavage started at 7days after implantation (day 0). Tumor size was measured twice per week. Values are mean ± SD (*n* = 6/group); *, *P* < 0.05, NC PF group versus sf-RON PF group. **B.** 24 days after the start of treatment, the mice were euthanized. The weight of the tumors in each group was measured. Values are mean ± SD (*n* = 6/group); *, *P* < 0.05, NC PF group versus sf-RON PF group. **C.** Tumor tissue from xenografts treated for 24 days with the indicated drug regimens was evaluated by immunohistochemical staining for *p*-ERK and Ki67. Representative images as indicated; Original magnification, x 400, Scale bar, 50 μm. **D, E, F.** PF significantly reduced pulmonary metastatic nodules in NC PF group, but not in sf-RON PF group. D, Representative lungs from each group as indicated. E, The number of pulmonary metastatic nodules in each group was calculated. Values are mean ± SD (*n* = 6/group); ***, *P* < 0.001, NC PF group versus NC vehicle group. F, Representative results of histological examination of mouse lungs for metastatic nodules in 4 groups. Left panel, Original magnification, x 100, Scale bar, 200 μm; right panel, Original magnification, x 400, Scale bar, 50 μm.

In order to confirm causal relationship between upregulation of sf-RON and PF resistance *in vivo*, firstly, xenograft tumors on immunocompromised mice were established with sf-RON overexpressed and NC GTL-16 cells. Mice were treated daily with vehicle (normal saline) or PF (4 mg/kg) by oral gavage 7days after the implantation of tumor fragments. After treating for 24 days, the tumor volume percent (%) inhibition value reached 85.7% in the NC group and 70.2% in the sf-RON group, (Figure 5A). The average tumor weight was heavier in the sf-RON-PF group than in the NC-PF group (average tumor weight 0.25 ± 0.05 g vs. 0.14 ± 0.09 g; *n* = 6; *P* = 0.039; Figure 5B), while the tumor weights in the sf-RON-vehicle group and NC-vehicle group were comparable, with no statistical differences (average tumor weight 0.53 ± 0.11 g vs. 0.54 ± 0.12 g; *n* = 6; *P* = 0.47; Figure 5B). The extent of phosphorylated ERK and Ki67 in tumor xenografts was examined in formalin-fixed paraffin-embedded tumor sections (Figure 5C). *P*-ERK was totally suppressed by PF in the NC group, while it was present in samples from the sf-RON-PF group. There were fewer cells with Ki67 staining in the NC-PF group than in the sf-RON-PF group.

Secondly, an *in vivo* murine experimental metastasis assay was performed by using sf-RON transformed GTL-16 cells and NC cells. In this assay, following injection of 1 × 10^6^ cells into the caudal vein of nude mice, animals were treated daily with vehicle (normal saline) or PF (1 mg/kg) by oral gavage for 45 days later. The results showed that the number of pulmonary metastatic nodules decreased significantly in the NC-PF group (*p* < 0.001), while the number of pulmonary metastatic nodules in sf-RON-PF group and sf-RON-vehicle group was similar (*p* = 0.177) (Figure 5D and 5E). Figure 5F showed representative images of the primary nodules of 4 groups. These results suggest that overexpression of sf-RON sustained cell proliferation and migration, which contributed to PF resistance *in vivo*.

### Sf-RON is frequently up-regulated in human GC samples

**Figure 6:**
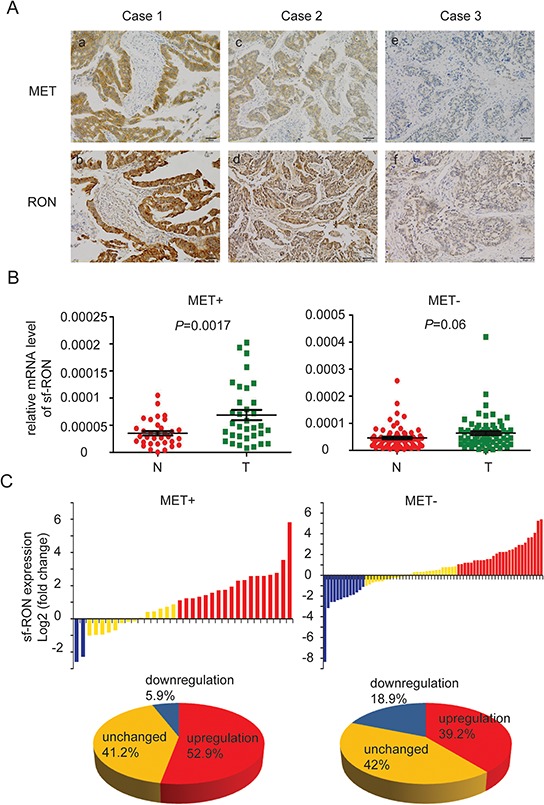
Expression of sf-RON was up-regulated in GC **A.** co-expression of RON and MET in human GC. Representative images showed RON and MET co-expressed in human GC samples. (a) and (b) indicated strong positive expression of MET and RON in the same GC tissue; (c) and (d) indicated positive expression; (e) and (f) indicated negative expression. Original magnification, x 200. Scale bar, 50 μm. **B.** Upregulation of sf-RON correlated with MET status of GC. The expression levels of sf-RON in 103 paired GC and matched non-tumor tissues were determined by q-PCR. The relative mRNA level of sf-RON in GC was compared with that in paired non-tumor tissues. Statistical analysis of differences between the two groups was performed by paired Student's *t*-test and *P* < 0.05 was considered statistically significant. **C.** sf-RON was up regulated in most MET-positive GCs. The data are expressed as the log_2_ fold change (ΔCt [GC/Non]). Significant upregulation of sf-RON expression in paired tumor/non-tumor samples was defined as a log_2_ fold change >1. The pie chart displayed the proportions of GC samples showing upregulation (red), downregulation (blue), and unchanged (yellow).

The above results demonstrated that activation of sf-RON signaling pathways confer resistance to MET inhibitor. To further validate the clinical significance of these findings, we analyzed expression of both transcripts of RON and FGFR2 in GC tissue samples, especially in MET+ samples. Firstly, the expression of MET, RON, and FGFR2 were assessed by immunohistochemistry (IHC) in 132 primary GC surgical samples. RON and FGFR2 were found to be highly expressed in 70.5% (93/132) and 19.7% (26/132) of specimens, respectively. There were more RON-positive samples in patients with MET+ tumors than in those with MET-negative tumors (94.7% vs. 66.4%; *P* = 0.013; Figure 6A and Table 1). While the positive expression rate of FGFR2 in MET+ cases was similar to that in MET-negative cases (26.3% vs. 30.8%; *P* = 0.532; Table 1).

**Table 1:** Association of RON, FGFR2, and MET expression status

No. of Patients (%)	MET			*P* value
	Negative	Positive	Total	
RON				
Negative	38(33.6)	1(5.3)	39(29.5)	0.013
Positive	75(66.4)	18(94.7)	93(70.5)	
FGFR2				
Negative	92(69.2)	14(73.7)	106(80.3)	0.532
Positive	21(18.6)	5(26.3)	26(19.7)	

Fisher's Exact test for all analyses.

Considering that sf-RON could not be distinguished by IHC, q-PCR was used to detect this isoform in GC cell lines and another 103 GC samples with specific primers. The status of MET expression in each sample was assessed through IHC (data not shown). Through statistical analysis, we found that the mRNA level of sf-RON was pervasively higher in GC cell lines compared with the immortality gastric cell line GES-1 (Supplementary Figure S5A). Moreover, the expression levels of the sf-RON in the tumor tissues significantly increased compared with those in adjacent non-tumor tissues, especially in MET+ samples (*p* = 0.0017) (Figure 6B and Supplementary Figure S5B). Then we compared the relative expression levels of sf-RON between the paired tumor and adjacent non-tumor tissues. Significant upregulation of sf-RON expression in paired GC/non-tumor samples was defined as a log_2_ fold change > 1 (i.e., 2-fold), and downregulation was defined as a log_2_ fold change < −1. As shown in Supplementary Figure S5C, in the total 103 GC samples, the proportion of specimens with sf-RON upregulation was 45.6%, the proportion of specimens with sf-RON downregulation was 14.7%, and the expression level of sf-RON was unchanged in the rest 41.7% ones. Furthermore, we found out that in MET+ GCs, the proportion of specimens with sf-RON upregulation (52.9%) was much higher than that with sf-RON downregulation (5.9%), while the proportion of specimens with sf-RON upregulation in MET- GCs was 39.2% (Figure 6C).

## DISCUSSION

Although MET kinase is regarded as one of the most promising therapeutic targets in gastric cancer, there are still a considerable number of MET+ patients who do not respond to anti-MET agents. Elucidating the mechanisms of intrinsic resistance to anti-MET therapy will be of great value in implementation of more efficient drug combination strategies. Through a siRNA library screening that targeted most genes encoding RTKs and subsequent validation, we found that knockdown of 4 RTK receptors (FGFR2, RON, EPHA1, and RYK) via siRNA sensitized MET-addicted tumor cells to PF, while b-FGF-induced activation of FGFR2 conferred resistance to the PF. Consistent with our work, a previous study observed that MET-addicted cancer cells could be rescued by ligands of FGFR from crizotinib sensitivity [13]. However, treating with ligands of the other 3 receptors failed to cause PF resistance. We speculated that ligands stimulation may not induce these RTKs pathways activation, with the treatment of MET inhibitor.

Of the 3 novel finding hits, we focused on RON pathway. It have been proved that RON actively crosstalks with MET, and is essential to support the oncogenic phenotype of MET-addicted cancer cells [18, 33]. Our data confirmed that, in *MET*-amplified GC cells, full-length RON was highly phosphorylated and could form heterodimers with MET. Phosphorylation of fl-RON was quenched by inhibition of MET via either specific TKI or siRNA. In the presence of MET inhibitor, MSP failed to reactivate fl-RON and downstream signaling. As a result, MSP was not able to rescue cancer cells from PF sensitivity. These findings imply that the activation of fl-RON strongly relies on the status of MET in MET-addicted cells. Thus, MSP induced activation of fl-RON pathway may be not involved in unresponsiveness to MET inhibitor.

Other than MSP induced dimerization and phosphorylation, aberrant activation of RON in cancer cells can also be achieved through overexpression of wild-type RON, and generation of activated mutations or isoforms [16]. Sf-RON, as a truncated transcript derived from the human *RON* gene, lacks the extracellular domains of this protein. Different from fl-RON, sf-RON proteins form disulfide-linked dimers automatically and constitutively phosphorylated, independently of MSP [34, 35]. Sf-RON has been detected in several types of cancer, and drives tumor progression [19]. Here, we provide evidence that in MET-addicted GC cells, the level of phosphorylated sf-RON is barely influenced by MSP or PF. And for the first time, we observed that the expression levels of sf-RON in the GC tissues significantly increased compared with those in adjacent non-tumor tissues. Moreover, in more than 50% of MET+ GC samples the mRNA level of sf-RON was upregulated.

Because of constitutive kinase activity, sf-RON is thought to play an important role in aggressive behaviors [19]. Our data revealed that upregulation of sf-RON in MET-addicted tumor cells attenuated PF-induced suppression of cell proliferation and migration. Similar to MET, RON signalling is classically mediated by MEK/ERK and PI3K/AKT pathways [16]. In the present study, we found that sf-RON maintain the activation of theses downstream signaling molecules, which could be inhibited via PF in control cells. These findings indicate that through generation of activated truncated isoform, RON pathway serves as a signaling compensatory mechanism, maintaining the growth and migration of PF-resistant cancer cells.

Rational combination strategy that simultaneously inhibits multiple targets or pathways may contribute to conquer drug resistance and improve efficacy of targeted therapeutics. Ichiro et al. have reported that dual inhibition of MET and RON signaling pathways by small-molecule inhibitor LY2801653 achieved dramatic antitumor effects in non-small cell lung cancer [36]. Here, we found that PF resistance induced by upregulation of sf-RON or activation of FGFR2 could be overcome by co-targeting MET with responsible bypass RTKs. Our observations might contribute to improve the efficacy of MET-targeted agents. It is worth noting that activation of sf-RON and FGFR2 pathways both reactive the downstream ERK signaling, which is originally suppressed by PF. MEK/ERK pathway is also considered as crucial node in RAF kinase conferred resistance to MET inhibition [37, 38]. All of these observations emphasize the important role of MEK/ERK pathway in overcoming resistance to anti-MET therapy.

In conclusion, our findings indicate that sf-RON signaling is implicated in the unresponsiveness of MET+ gastric cancer to MET inhibitors. Considering the high proportion of sf-RON overexpresson in MET+ gastric cancer samples, activation of this pathway might serve as a crucial signaling compensatory mechanism that attenuates the efficacy of anti-MET therapy. Further clinical studies are required to evaluate the efficacy of combination strategies co-targeting MET and RON signaling pathways on patients with GC.

## MATERIALS AND METHODS

### Cell lines

GC cell lines MKN-45 and MKN-28, and immortalized human gastric epithelial cell line GES-1, were obtained from 3D Biopharm Biotech Co. Ltd. (Shanghai, China). NCI-N87, HGC-27, AGS, and SGC-7901 cell lines were obtained from the Cell Bank of Type Culture Collection of Chinese Academy of Sciences (Shanghai, China). SNU-216 and GTL-16 cell lines were gifts from the Medical College of Xiamen University (Xiamen, China) and AstraZeneca China R&D Center (Shanghai, China). EBC-1 cell line was obtained from COBIOER BIOSCIENCES Co. Ltd (Nanjing, China). Cell lines were tested and authenticated by short tandem repeat DNA profiling analysis before execution of the experiments. Cells were cultured in Minimum Essential Medium (HGC-27), F12K medium (AGS), Dulbecco's Modified Eagle's Medium (GTL-16), Eagle's Minimum Essential Medium (EBC-1) or Roswell Park Memorial Institute 1640 medium containing 10% fetal bovine serum (Gibco, Carlsbad, CA, USA) and 1% penicillin-streptomycin (Invitrogen, Carlsbad, CA, USA) at 37°C in a humidified atmosphere with 5% carbon dioxide.

### Drug preparations

All tyrosine kinase inhibitors (TKIs) used were purchased from Selleck Chemicals (Houston, TX, USA). Recombinant human basic fibroblast growth factor (b-FGF) was purchased from PeproTech (Rocky Hill, NJ, USA). Recombinant human macrophage-stimulating protein (MSP), ephrinA1, and Wnt5a were from R&D Systems (Minneapolis, MN, USA). Compounds were dissolved in 100% dimethylsulfoxide (DMSO) (Sigma-Aldrich, St Louis, MO, USA) and diluted with culture medium to the desired concentration, with a final DMSO concentration <0.2% (v/v). DMSO was also added to control cells in culture.

### siRNA library screening and validation

Screening was conducted using a siRNA library (QIAGEN, Hilden, Germany) containing 240 siRNAs with 4 unique siRNA species targeting each of 60 distinct RTKs. Scrambled control siRNA was used as the negative control (NC). On day 1, siRNA (final concentration = 5 nM) was first added to 96-well plates and incubated with HiPerFect Transfection Reagent (QIAGEN, Hilden, Germany) to allow formation of transfection complexes, then MKN-45 cells were seeded at 2500 per well. On day 2, cells were treated with PF04217903 (PF) 0.012 μM or 0.2% DMSO alone (vehicle). Cell viability was assessed 4 days later using Cell Counting Kit-8 (CCK-8) Cell Viability Assay following the manufacturer's protocol. The assay was performed in triplicate. To evaluate gene targets that increase PF sensitivity or resistance, the sensitivity index (SI) was calculated as described [21, 39]. In brief, the effect of siRNA compared to negative siRNA control was designated Rc/Cc, and effect of the drugs on control-transfected cells was designated Cd/Cc. The expected combined effect of siRNA and drug on cell viability could be calculated by Rc/Cc × Cd/Cc. The Observed combined effects of drug and siRNA on cell viability compared to negative siRNA control was designated Rd/Cc. Then, an index of antagonism or sensitivity (SI) for each siRNA was calculated by the Observed combined effect minus the Expected combined effect: SI = (Rc/Cc*Cd/Cc) − (Rd/Cc). A positive SI score indicates a sensitizing effect and a negative SI score indicates antagonism to the treatment, an additional criteria that Rd/Cd < 0.95 (for sensitizing siRNAs) was employed for hit selection. SI scores was ranked and those in the upper quartile were considered as conferring a strong drug-sensitizing phenotype. Another 4 distinct siRNA species (QIAGEN) targeting each gene were used to validate hits from the initial screen. The transfection process was consistent with that in the screening. Twenty four hours after transfection, cells were treated with vehicle or a dose range of PF (0.008–0.024 μM). Cell viability was assessed 4 days later through CCK-8 Assay.

### Cell viability and colony formation assay

Cell line sensitivity to the indicated treatment was determined through Cell Viability Assay. In brief, cells were seeded at 2,500 cells per well in 96-well plates and incubated overnight. Cells were then treated with increasing concentrations of the indicated drugs and other agents for 72 hours. Treatments at each concentration were carried out in 6 replicate wells and repeated 3 times. Cell viability was determined using the CCK-8 according to the manufacturer's instructions. Colony formation assay was conducted as previously described [39]. Briefly, MKN-45 cells were seeded at 1 × 10^4^ cells per well in triplicate in 12-well plates and cultured in the absence or presence of the drug and ligands for 10 days. After fixing the cells with 4% paraformaldehyde, cell clones were stained with 0.1% crystal violet.

### *In vitro* migration assays

Cell migration was analyzed by using a BD Falcon Cell Culture Insert System (BD Biosciences, San Jose, CA, USA) with 8-μm pores. For motility assays, 6 × 10^4^ GTL-16 cells in 300 μL of serum-free medium were seeded into upper inserts, with 600 μL of 10% serum medium to the lower chamber. After treating with indicated drugs and agents for 48 hours, cells were fixed with 0.5 mL of 4% paraformaldehyde. Then, each well was washed 3 times with 1× PBS, and stained with 0.6 mL of 0.1% crystal violet solution. After removing the cells on the upper chamber using a cotton swab, the number of cells was counted at 5 fields per membrane at 200x magnification from each group of 3 independent experiments.

### Lentivirus production and transduction

Virus packaging was performed in human embryonic kidney (HEK) 293T cells after cotransfection of pWPXL-sf-RON or empty vector with the packaging plasmid psPAX2 (Addgene plasmid 12260; Didier Trono Lab, Cambridge, MA, USA) and the envelope plasmid pMD2.G (Addgene plasmid 12259, Didier Trono Lab) using Lipofectamine^®^ 2000 (Invitrogen, Carlsbad, CA, USA). Viruses were harvested 48 hours after transfection. Target cells, including MKN-45 and GTL-16 were infected with filtered lentivirus in the presence of 6 mg/mL Polybrene^®^ (Sigma-Aldrich, St Louis, MO, USA).

### Western blot and antibodies

Cells were lysed in radioimmunoprecipitation assay buffer (Biyotime, Shanghai, China) supplemented with complete protease inhibitor cocktail (Roche, Basel, Switzerland). Protein concentrations were determined using the BCA protein assay kit (Biyotime, Shanghai, China). Western blot was carried out with 30 μg total proteins and antibodies against MET, *p*-MET, *p*-FGFR2, AKT, *p*-AKT, ERK, *p*-ERK kinase, β-actin and GAPDH (Cell Signaling Technology, Cambridge, MA, USA), RON and *p*-RON (Santa Cruz Biotechnology, CA, USA), and FGFR2 (R&D Systems, Minneapolis, MN, USA). Blots were probed with the indicated primary antibodies, then incubated with the horseradish peroxidase-conjugated secondary antibody and detected by enhanced chemiluminescence reagent (Pierce, Rockford, IL, USA).

### Immunoprecipitation

The experiment was performed by using Pierce™ Classic Magnetic IP/Co-IP Kit (Pierce, Rockford, IL, USA), according to the manufacturer's instructions. In brief, cells were lysed in buffer (pH 7.4) containing 0.025 M Tris, 0.15 M NaCl, 0.001 M EDTA, 1% NP40, 5% glycerol, protease and phosphatase inhibitor cocktails. 500 μg total protein were immunoprecipitated overnight at 4°C with the antibody against MET (Cell Signaling Technology, Cambridge, MA, USA). Add the antigen sample/antibody mixture (Section B) to the tube containing pre-washed magnetic beads and incubate at room temperature for 1 hour with mixing. The immunocomplexes were eluted by Elution Buffer, and immunoblotted to detect RON. Irrelevant IgG served as a negative control.

### Xenograft experiments

Male athymic nude mice, aged 6 weeks, were purchased and raised under specific pathogen-free conditions at Shanghai Slac Laboratory Animal Co. Ltd. (Shanghai, China). All animal work must have been conducted according to relevant national and international guidelines. Firstly, Stable GTL-16 cell lines transfected with empty vector (GTL-16 NC) or sf-RON (GTL-16 sf-RON) were subcutaneously implanted into the right flanks region of the mice (10^7^ cells in 0.1 mL PBS), respectively. Once the volume of the tumor xenograft reached approximately 300–500 mm^3^, tumors were further established in the nude mice (*n* = 6/group) by subcutaneous implantation of tumor fragments (~2 × 2 × 2 mm) obtained from donor mice. Treatment started 7days after implantation (day 0) with either PF (4 mg/kg, daily) or vehicle (normal saline, daily) by oral gavage for 24 days. Tumor volumes were determined from caliper measurements of tumor length (L) and width (W) according to the formula LW^2^/2. Both tumor size and body weight were measured twice per week. Percent inhibition values were calculated as: 100 × (1−((PF_final day_ −PF_day 0_)/(vehicle_final day_− vehicle_day 0_))). Tumor volumes were analyzed using one-way analysis of variance (ANOVA). At the end of the treatment period, mice were humanely euthanized and the tumors were weighed and fixed in 10% buffered formalin for immunostaining. Tumor weights were compared using *t* test.

### *In vivo* metastasis assays

For *in vivo* murine Experimental metastasis assays, cells (1 × 10^6^ per mouse) were injected into the tail veins of nude mice, followed by treating animals with either PF (1 mg/kg, daily) or vehicle (normal saline, daily) by oral gavage. Six weeks later, all of the mice were sacrificed and the lungs were dissected and processed for standard histological studies. For histological analysis, the mouse organs were fixed in 10% formalin and paraffin-embedded and 4 serial sections were obtained from each sample. The sections were stained with hematoxylin and eosin (H&E) and analyzed for the presence of metastases. The number of pulmonary metastatic nodules between PF-treating group and vehicle-treating group was compared using *t* test.

### Patients and specimens

All primary gastric adenocarcinomas specimens were obtained by surgical resection between 2007 and 2010 at Shanghai Cancer Center, Fudan University, Shanghai, China. Samples were acquired after informed consent was given, under the protocol approved by the Shanghai Cancer Center research ethics committee. Tissue microarrays were constructed in collaboration with Shanghai Biochip (Shanghai, China), as described [39]. Additionally, a set of 103 fresh frozen GC samples was analyzed for expression of sf-RON and full-length RON (fl-RON).

### Immunohistochemistry and scoring system

Paraffin embedded GC tissue sections were subjected to immunohistochemical assays using a MaxVision^TM^ HRP-Polymer Detection System (Maixin, Fuzhou, China), as described previously [39]. Primary antibodies against MET (Roche, Basel, Switzerland), RON (Santa Cruz Biotechnology, Santa Cruz, CA, USA), and FGFR2 (R&D Systems, Minneapolis, MN, USA) were used to assess the expression of MET, RON, and FGFR2 in 132 primary GC surgical samples. Primary antibodies against *p*-ERK (Cell Signaling Technology) and Ki67 (Dako, Denmark) were used on GC xenograft sections.

Slides were independently evaluated by 2 investigators who were blinded to the clinical information. The intensity of MET, RON, and FGFR2 membrane immunostaining and prevalence of these intensities in tumor cells were evaluated. Staining intensity (0 none, 1+ weak, 2+ moderate, 3+ strong) and percentage of cells staining were independently scored. Samples that scored at least 2+ in at least 50% of tumor cells were regarded as positive for each marker [40].

### RNA extraction and reverse transcription-polymerase chain reaction

Total RNA was extracted from tumor tissues using Trizol Reagent (Invitrogen, Carlsbad, CA, USA), and reversely transcribed through PrimeScript^TM^ reverse transcription-polymerase chain reaction (RT-PCR) kit (TaKaRa, Shiga, Japan) according to the manufacturer's instructions, as described [41]. mRNA expression levels were quantified with SYBR Premix Ex Taq kit (TaKaRa, Japan) on a 7900 Real-Time PCR System with SDS 2.3 software (Applied Biosystems) at the recommended thermal cycling settings: one initial cycle at 95°C for 10 s followed by 40 cycles of 5 s at 95°C and 31 s at 60°C. To examine sf-RON transcript expression, specific primers were designed as follows: sense: 5′- TATATTGGGCTGGGCGCTGTGG-3′ and antisense: 5′- TACAATGGGGCACCATCCTG-3′. The expression level of sf-RON were normalised to the internal reference gene 18s rRNA (sense, 5′-GTAACCCGTTGAACCCCATT-3′ ; antisense, 5′-CCATCCAATCGGTAGTAGCG-3′) [42].

### Statistical analysis

Statistical analyses were performed with the Statistical Package for the Social Sciences version16.0 (SPSS Inc., Chicago, IL, USA) or with GraphPad Prism version 5.0 (GraphPad Software, La Jolla, CA, USA). Quantitative variables were analyzed by Student's *t* test or one-way ANOVA with Bonferroni post-test. Fisher's exact test was used to compare qualitative variables. Differences between cell viability and growth curves were analyzed by two-way ANOVA followed by Bonferroni's multiple comparison test. Two-tailed *P* < 0.05 was considered statistically significant.

## SUPPLEMENTARY MATERIALS AND METHODS FIGURES AND TABLE


