# Supplementary material for: Identification of short-form RON as a novel intrinsic resistance mechanism for anti-MET therapy in MET-positive gastric cancer
Source: Oncotarget. 2015 Oct 26;6(38):40519–34. doi: 10.18632/oncotarget.5816 (PMC4747350; doi:10.18632/oncotarget.5816)
Supplement: Supplementary file 1 [file oncotarget-06-40519-s001.pdf]

SUPPLEMENTARY MATERIALS AND METHODS

Cell viability

Cell line sensitivity to the indicated treatment was determined through Cell Viability Assay. In brief, cells were seeded at 2,500 cells per well in 96-well plates and incubated overnight. Cells were then treated with increasing concentrations of the indicated drugs and other agents for 72 hours. Treatments at each concentration were carried out in 6 replicate wells and repeated 3 times. Cell viability was determined using the CCK-8 according to the manufacturer's instructions. The half maximal inhibitory concentration (IC_{50}) was determined by using the non-linear regression model in GraphPad Prism version 5.0 (GraphPad Software, La Jolla, CA, USA).

SUPPLEMENTARY FIGURES AND TABLES

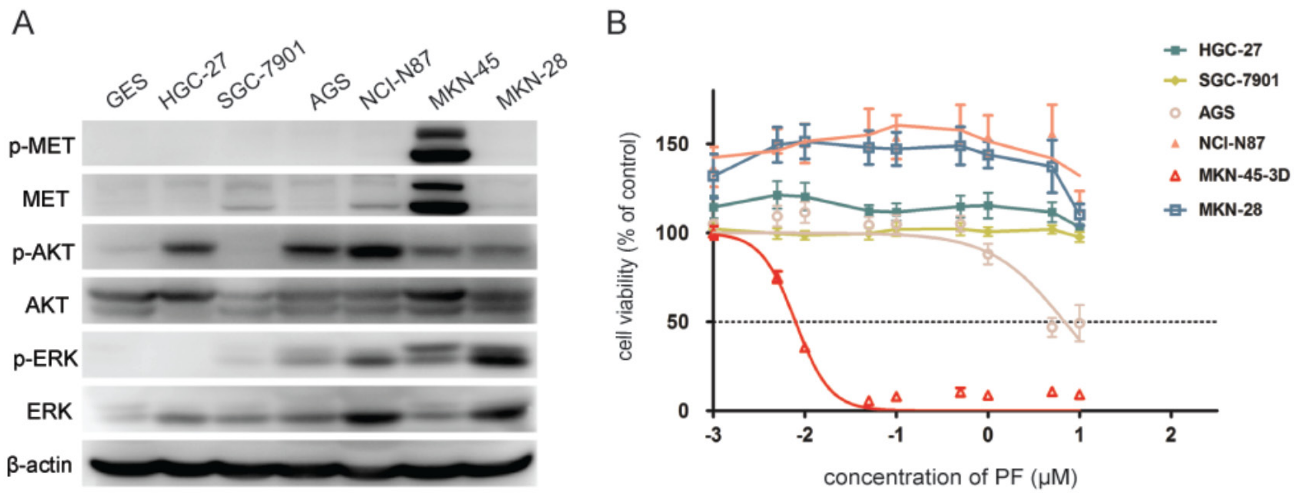

Supplementary Figure S1: MET-positive MKN-45 cells displayed responsiveness to PF compared with MET-negative cells. A. MKN-45 human GC cells showed MET overexpression and activation. Protein lysates were collected from all 6 GC cell lines and 1 immortalized gastric epithelial cell line GES-1, and analyzed for MET expression and activation, as well as key downstream components. β-actin served as a loading control. **B.** The MKN-45 cell line showed more responsiveness to PF in all 6 GC cell lines. Cells were treated with PF at the indicated concentrations, and viable cells were measured by CCK-8 cell-proliferation assay after 72 hours. The percentage of viable cells is shown relative to untreated controls. Data points indicate average of replicates of 6 and bars indicate SD.

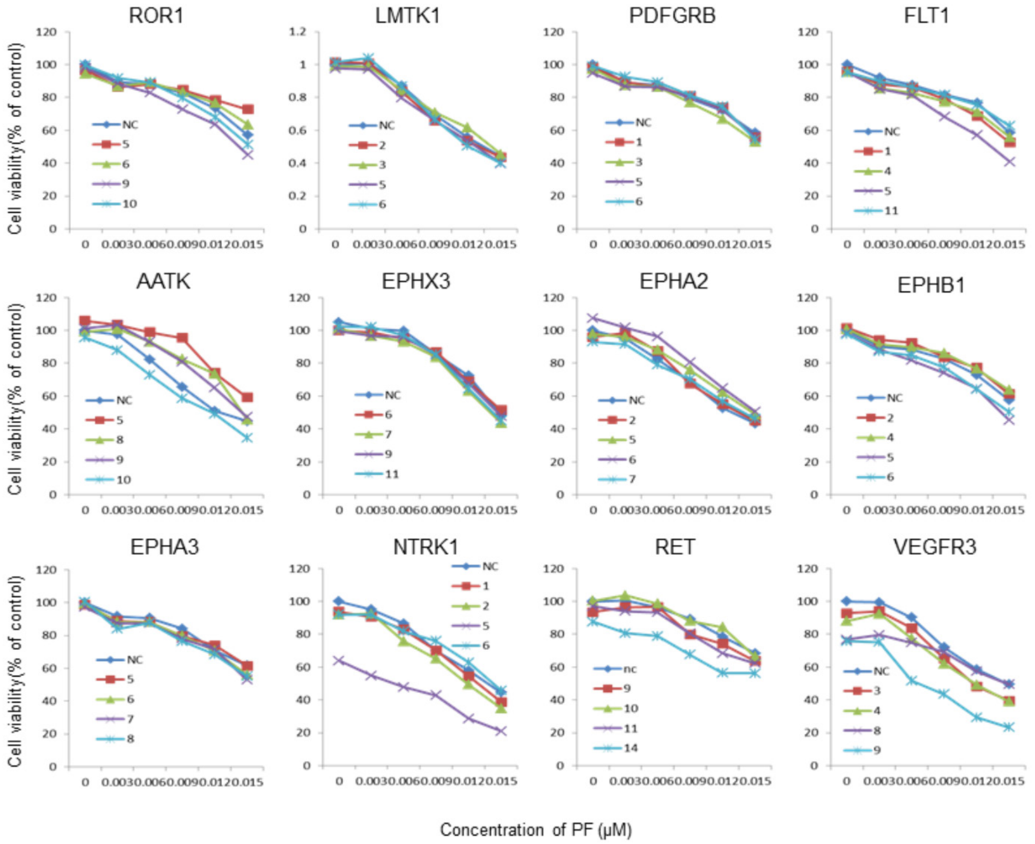

Supplementary Figure S2: Validation of synthetic lethality hits. In these 16 candidates, ROR1, LMTK, PDGFRB, FLT1, AATK, EPHX3, EPHA2, EPHB1, EPHA3, NTRK1, RET or VEGFR3 gene silencing showed no significant enhancement of PF effectiveness. MKN-45 cells were transfected with non-targeting control (NC) siRNA and 4 siRNA species targeting each gene. Cell viability was measured using the CCK-8 cell-proliferation assay after 4 days of drug exposure. The percentage of viable cells is shown relative to untreated non-targeting controls. Values are the mean of 3 assays.

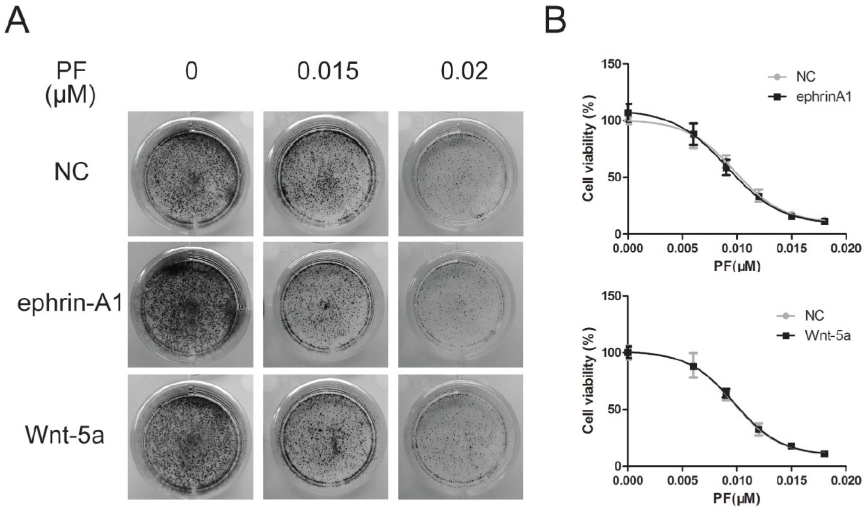

Supplementary Figure S3: Ligands of EPHA1, and RYK hardly influenced PF response in MET-addicted GC cell lines. A. Colony formation assays showed that compared with control group, supplement of ephrinA1 or Wnt-5a resulted in no increase of PF-resistant MKN-45 cell clones. **B.** ephrinA1 or Wnt-5a failed to rescue the inhibition of cell viability by PF. GTL-16 cells were exposed to increasing concentrations of PF (0-0.02 μM) with and without ephrinA1 (100 ng/mL) or Wnt-5a (50 ng/mL). Cell viability was measured using the CCK-8 cell-proliferation assay after 3 days of drug exposure. Data points indicate average of 3 replicates and bars indicate SD.

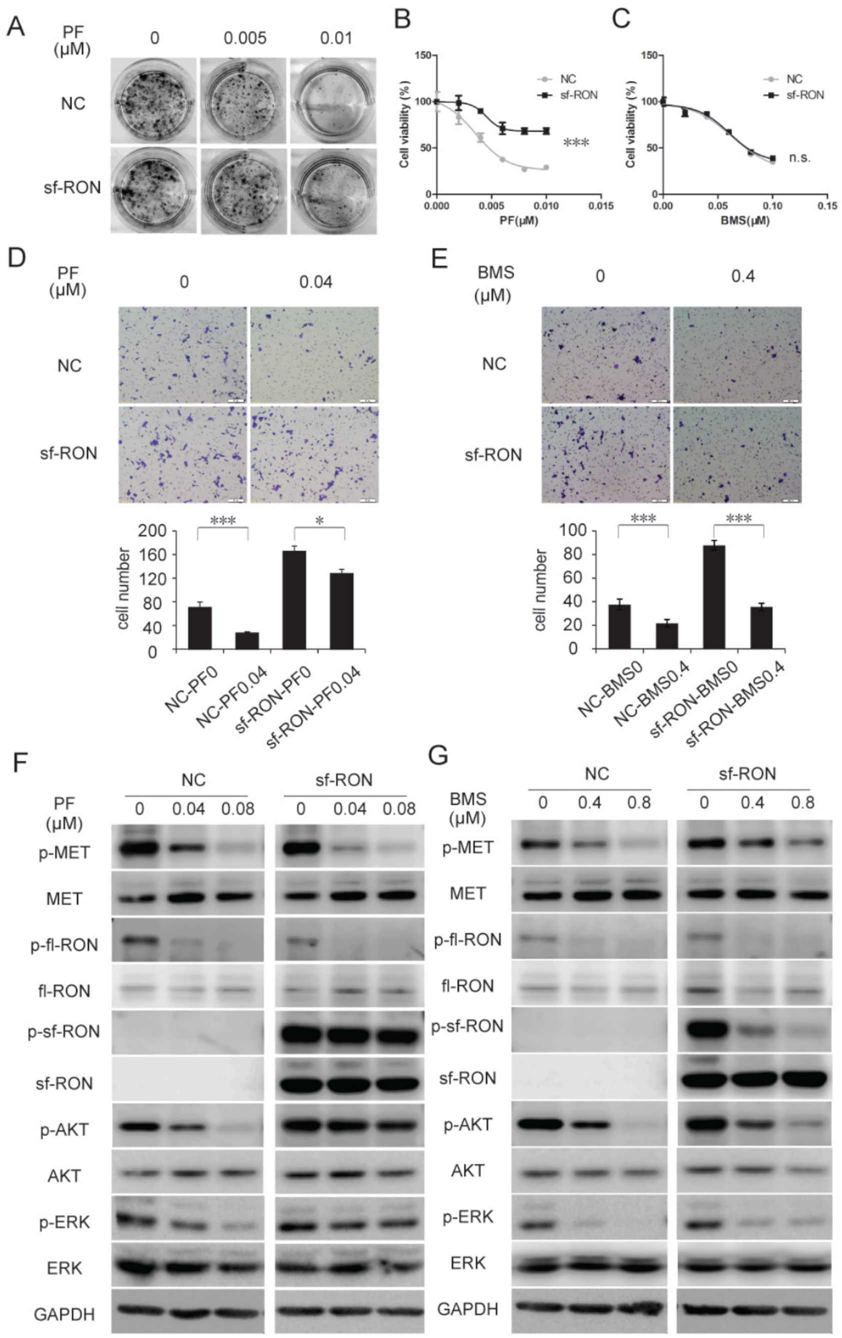

Supplementary Figure S4: Upregulation of sf-RON attenuated PF-induced inhibition of cell proliferation and motility. A. and **B.** sf-RON induced PF resistance in EBC-1 cell lines. A, Colony formation assays showed that PF-resistant cell clones in the sf-RON group were greater than those in the NC group. B, cells were treated with increasing doses of PF (0-0.024 μM), followed by CCK-8 cell-proliferation assay at 72 hours. **C.** MET/RON dual inhibition suppressed viability of cells with sf-RON overexpression effectively. Cells were treated with BMS (0–0.5 μM), followed by CCK-8 cell-proliferation assay at 72 hours. **D.** and **E.** BMS, but not PF, abrogates sf-RON-induced extra cell motility. Transwell migration assays of EBC-1 cells were performed with the treatment of 0.04 μM PF (D) or 0.4 μM BMS (E) for 48 hours. Representative images of migration assays and statistics in bar graphs as indicated. Bars, 50 μm. Values are the mean ± SD of 3 assays; ***, $P < 0.001$, n.s., no statistical signficance. **F.** sf-RON confers PF resistance by restoring AKT and ERK activation. **G.** BMS total blocked phosphorylation of MET/RON and downstream signaling. Cells in the NC group and sf-RON group were pretreated with PF (F) or BMS (G) for 6 hours before whole cell lysates were collected. Western blot analysis was conducted to examine the status of *p*-RON, *p*-MET, *p*-AKT, and *p*-ERK.

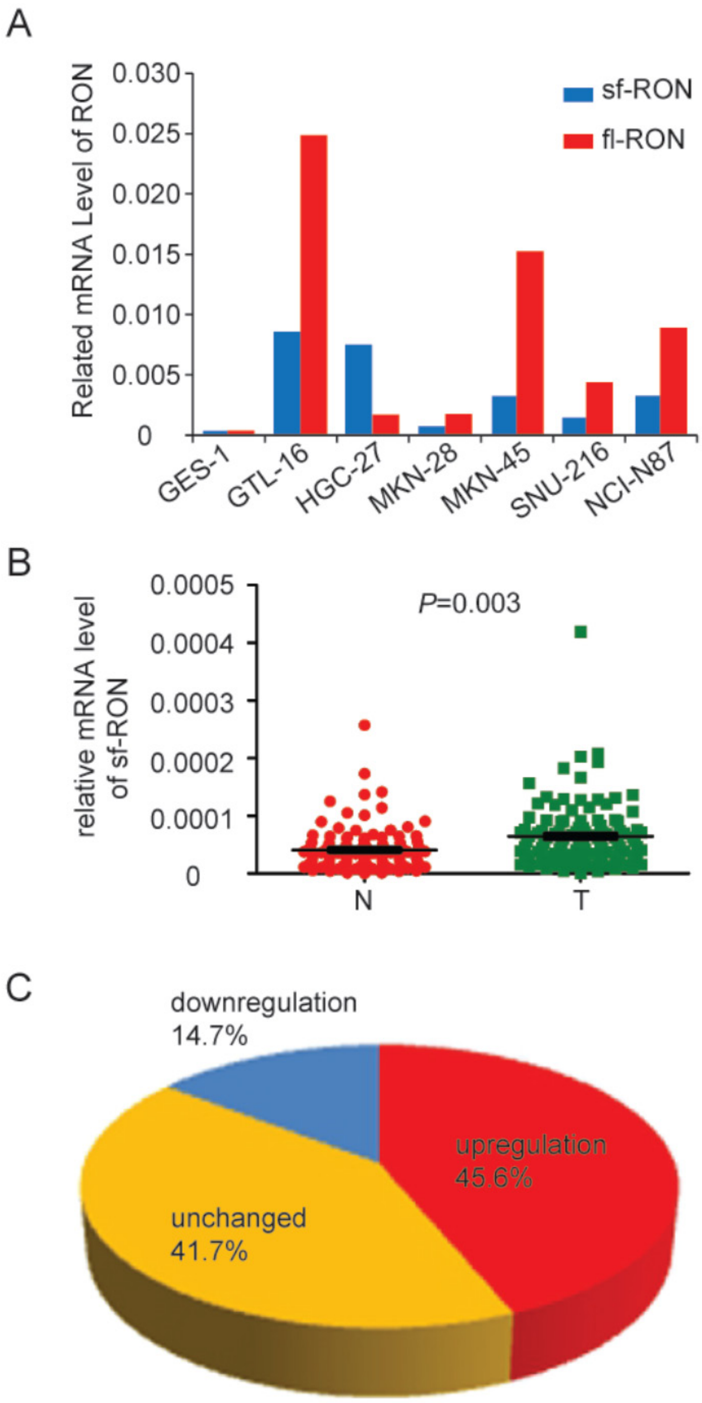

Supplementary Figure S5: Expression of sf-RON was up-regulated in GC. A. mRNA level of RON is upregulated in GC cell lines. The mRNA level of sf-RON and fl-RON were evaluated in 6 GC cell lines and GES-1 cells respectively. **B.** and **C.** sf-RON was up-regulated in GC samples. B, the expression levels of sf-RON in 103 paired GC and matched non-tumor tissues were determined by q-PCR. The relative mRNA level of sf-RON in GC was compared with that in paired non-tumor tissues. Statistical analysis of differences between the two groups was performed by paired Student's *t*-test and $P < 0.05$ was considered statistically significant. C, the pie chart displayed the proportions of GC samples showing upregulation (red), downregulation (blue), and unchanged (yellow).

Supplementary Table S1: Clinicopathological characteristics of patients

Features		No. of Patients	%
age			
	≤58	68	51.5
	>58	64	48.5
sex			
	female	31	23.5
	male	101	76.5
Histologic grade			
	G1 Well differentiated	1	0.8
	G2 Moderately differentiated	18	13.6
	G3 Poorly differentiated	109	82.6
	G4 Undifferentiated	4	3
vascular invasion			
	yes	78	59.1
	no	54	40.9
nerve invasion			
	yes	75	56.8
	no	57	43.2
AJCC TNM stage			
	I	6	4.5
	II	34	25.8
	III	80	60.6
	IV	12	9.1

AJCC, American Joint Committee on Cancer.
